# Efficacy of slow-release collar formulations of imidacloprid/flumethrin and deltamethrin and of spot-on formulations of fipronil/(s) - methoprene, dinotefuran/pyriproxyfen/permethrin and (s) –methoprene/amitraz/fipronil against *Rhipicephalus sanguineus* and *Ctenocephalides felis felis* on dogs

**DOI:** 10.1186/1756-3305-5-79

**Published:** 2012-04-22

**Authors:** Ivan G Horak, Josephus J Fourie, Dorothee Stanneck

**Affiliations:** 1 Department of Zoology and Entomology, University of the Free State, Bloemfontein, 9301, South Africa; 2 Department of Veterinary Tropical Diseases, Faculty of Veterinary Science, University of Pretoria, Onderstepoort, 0110, South Africa; 3 ClinVet International, P.O. Box 11186, Universitas, 9321, South Africa; 4 Bayer Animal Health GmbH, 51368, Leverkusen, Germany

**Keywords:** Fleas, Ticks, Efficacy, Imidacloprid, Flumethrin, Collar, Deltamethrin, Fipronil, Methoprene, Amitraz, Dinotefuran, Spot-on

## Abstract

**Background:**

Two studies evaluating the efficacy of an imidacloprid/flumethrin collar (Seresto®, Bayer Animal Health, IVP), a deltamethrin collar (Scalibor®, MSD, CP1), a fipronil/(s)-methoprene spot-on (Frontline Combo®, Merial, CP2), a dinotefuran/pyriproxyfen/permethrin spot-on (Vectra 3D®, Ceva, CP3) and an amitraz/fipronil/(s)-methoprene spot-on (Certifect®, Merial, CP4/CP5) against repeated infestations with *Rhipicephalus sanguineus* and *Ctenocephalides felis felis* on dogs were conducted over periods of 226 days and 71 days respectively.

**Methods:**

The first study comprised 4 groups of treated dogs and one untreated control group, and the second 3 groups of treated dogs and one control group. Each group consisted of 8 dogs. All dogs were infested with ticks and fleas at regular intervals. Ticks were counted 6 h, 18 h or 48 h after infestations and fleas 24 h after infestations. Efficacies of the treatments were calculated by comparison with the untreated control groups using standard descriptive statistics.

**Results:**

The protective 48 h tick efficacy was 97.8% to 100% for the IVP (226 days), 69.3% to 97.4% for CP1 (170 days), 99.6% to 43.4% for CP2 (35 days) and 98% to 61.4% for CP3 (35 days).

The protective 18 h tick efficacy was 98% to 99.6% for the IVP (71 days), 100% to 86.5% for CP4 (29 days), 100% to 72.8% for CP4 after re-treatment (35 days) and 98.8% to 54.3% for CP5 (35 days).

The protective 6 h tick efficacy was 85.6% at Day 7 and 90.1% to 97.1% from Day 14 onwards for the IVP (70 days), 92.3% to 70.7% for CP4 (35 days), 97.5% to 65.2% for CP4 after re-treatment (35 days) and 95.1% to 51.8% for CP5 (35 days).

The protective 24 h flea efficacy was 99.5/90.9% to 100% for the IVP (71/226 days), 66.7% to 83% for CP1 (170 days), 100% to 88.5% for CP2 (35 days), 100% to 73.3% for CP3 (35 days), 100% to 98.7% for CP4 (35 days), 100% to 87.5% for CP4 after re-treatment (35 days) and 100% to 79.5% for CP5 (35 days).

**Conclusions:**

These data suggest that the long-term efficacy provided by a medicated collar that is effective, is a means to overcome the fluctuating efficacy of spot-on treatments resulting from a lack of pet owner re-treatment compliance, and consequently protect animals successfully against ectoparasites and probably vector-borne diseases.

## Background

Ticks and fleas are parasites of dogs practically world-wide. Although certain tick species are predominant on dogs in some regions, the brown dog tick, *Rhipicephalus sanguineus* is present on these animals in several regions and predominant in others [1-3]. On the other hand the cat flea, *Ctenocephalides felis felis* is widespread throughout most regions, and infests both dogs and cats [4,5]. The effective control of these ectoparasites not only alleviates the immediate distress caused to their hosts, such as itching, skin lesions and blood loss, but may also prevent the direct effects of infestation such as tick-induced paralysis and flea allergy dermatitis [6]. Furthermore, a reduction in parasite numbers will inevitably have an effect on the prevalence of the diseases that they transmit. It is also possible that chemicals that have a repellent, or a particularly rapid killing effect, could eliminate ticks and fleas before they can transmit vector-borne organisms with which they may be infected. For instance *R. sanguineus* is a vector of *Babesia canisBabesia vogeliBabesia gibsoniHepatozoon canis* and *Ehrlichia canis*, the causative organisms of tick-borne diseases that affect dogs in different regions of the world [7]. On the other hand *C. felis felis* is the intermediate host of the larval stage of the tapeworm *Dipylidium caninum* of dogs and the vector of the bacterium *Rickettsia typhi*, the organism responsible for murine typhus in humans [8].

Several chemicals, or combinations of chemicals, with acaricidal or insecticidal properties and which are appropriate and safe for treatment of domestic dogs and cats, have been formulated for application either orally, parentally, topically or as medicated collars [9,10]. Depending on the active ingredients of the chemicals or combinations of chemicals, they are effective against fleas or ticks, or both [11,12], and may in addition also be suitable for the treatment of lice and mites [13,14]. Some of these chemicals also have a persistent effect lasting for several weeks after their initial application [12], while others are effective for several months [15].

The aims of the present investigation were to evaluate the efficacies of various remedies against ticks and fleas on dogs. To this end two studies were performed during which the efficacies of five chemicals or combinations of chemicals were assessed against laboratory-induced infestations of *R. sanguineus* and *C. felis felis*. For the sake of clarity the methods applicable to both studies have been presented as an entity, and thereafter the experimental designs and results of each study are presented separately.

## Methods: general

The studies were conducted in South Africa and were parallel group-designed, randomised, unicentre, controlled efficacy studies and were performed on groups of eight dogs each. The dogs enrolled in the studies were sub-adult and adult male and female mixed-breeds weighing between 9.26 and 25.30 kg. They were maintained and handled with due regard for their welfare, and were acclimatized to the kennel environment seven to ten days prior to the commencement of the studies. The dogs were individually housed in pens in animal units that conformed to the South African National Standards (SANS 10386:2008 *The care and use of animals for scientific purposes*). Water was available *ad libitum* and an adequate amount of a commercial dog food towards their maintenance was provided daily.

A laboratory-bred strain of *R. sanguineus*, originating from France and subsequently maintained for at least 3 years on rabbits in South Africa, was used for infestation of the dogs. Adult ticks used for infestation were unfed, at least one week old, and of a balanced sex ratio (50% female: 50% male). Each dog was infested with 50 ticks on the days indicated in the respective experimental designs.

A laboratory-bred strain of *C. felis felis*, originating from Hanover University, Germany, and maintained on cats in South Africa for at least 2 years prior to the studies, was used for all infestations. Each dog was infested with approximately 100 unfed fleas of mixed sex on the days indicated in the respective experimental designs. At the time of infestation neither the ticks nor the fleas were placed near the collars, or on or near the site, or sites, where the spot-on treatments were or had been administered.

The time at which each animal was treated or at which it was infested with ticks was recorded. This was done to ensure that counting and removal of ticks were as close as possible to the specified target times (6 h ± 30 min, or 18 h ± 1 h, or 48 h ± 2 h post infestation or treatment). During the *in situ* counts, ticks were counted but not removed. Ticks were found by direct observation following parting of the hair coat and palpation over the whole skin surface of the dog. The same procedure was followed for the removal tick counts, but in addition the dogs were combed after the ticks had been removed to ensure that all ticks were found and counted. Ticks that were removed were classified according to the parameters listed in Table 1.

**Table 1:** Classification of ticks on their state of attachment and engorgement and whether they are alive or dead

**Category**	**Condition**	**Attachment status**
**1**	Alive	Unattached
**2**	Alive	Attached; unengorged*
**3**	Alive	Attached; engorged**
**4**	Dead	Unattached
**5**	Dead	Attached; unengorged*
**6**	Dead	Attached; engorged**

* no filling of the alloscutum evident.

** obvious or conspicuous filling of the alloscutum.

The time at which each animal was treated or at which it was infested with fleas was recorded. This was done to ensure that counting was as close as possible to the specified target times (48 ± 2 h post-treatment or 24 ± 2 h post-infestation). During counting a fine-toothed flea comb was used to recover fleas from the animal’s hair. Combing was performed by several strokes of the comb over each part of the dog’s skin surface, each time in the same direction and following the lie of the hair coat. Movement, from one part of the animal’s body to the next, was via strokes overlapping each other, so that no part of the skin surface was missed. After the completion of combing, the whole procedure was repeated so that all areas were combed at least twice. If necessary a third combing was performed until no live fleas were found.

The pre-treatment flea counts of each dog were used for ranking and group allocation purposes. The dogs were ranked within gender, in descending order of individual pre-treatment flea counts. Animal IDs were used to break ties. Within each gender, animals were then blocked and within each block dogs were randomly allocated to the various treatment groups.

Efficacy against ticks and fleas was calculated for each treatment group at each assessment day according to the formulas below. All efficacy calculations were performed on the arithmetic means of the tick or flea counts.

In accordance with the *Guidelines for the testing and evaluation of the efficacy of antiparasitic substances for the treatment and prevention of tick and flea infestation in dogs and cats;* EMEA/CVMP/005/2000- Rev.2: percent efficacy for the *in situ* tick counts was calculated as follows:

(1) Efficacy %=100 x  mc– mt/ mc, where

m_c_ = arithmetic mean number of live ticks (categories 1-3) on dogs in the untreated control group at a specific time-point.

m_t_ = arithmetic mean number of live ticks (categories 1-3) on dogs in the treated groups at a specific time-point.

Percent efficacy for the removal tick counts was calculated as follows:

(2) $$\text{Efficacy }\left(\%\right)=100\text{ x }\left(m_{c}–m_{t}\right)/m_{c},\text{ where}$$

m_c_ = arithmetic mean number of live ticks (categories 1-3) on dogs in the untreated control group at a specific time-point.

m_t_ = arithmetic mean number of live and dead ticks (categories 1-3 and 6) on dogs in the treated groups at a specific time-point.

Percent efficacy against fleas was calculated as follows:

(3) $$\text{Efficacy }\left(\%\right)=100\text{ x }\left(m_{c}–m_{t}\right)/m_{c},\text{ where}$$

m_c_ = arithmetic mean number of live fleas on dogs in the untreated control group

m_t_ = arithmetic mean number of live fleas on dogs in the treated groups.

Descriptive statistics (mean, minimum, maximum, standard deviation, CV%, arithmetic mean and median) on tick and flea counts on the various assessment days were also performed. SAS Version 8 (Release 8.02 TS Level 02 M0) was used for all the statistical analyses.

### Methods: study 1

This study was designed to ascertain the immediate and the long-term efficacy of a collar containing imidacloprid 10% and flumethrin 4.5% (w/w) (Seresto®, Bayer Animal Health) and a collar containing deltamethrin 4% (w/w) (Scalibor®, MSD) against *R. sanguineus* and *C. felis felis* on groups of experimentally infested dogs, compared to an untreated control group of dogs infested with the same ectoparasites. In addition a group of dogs treated with a spot-on formulation of fipronil 10% (w/v) and (s)-methoprene 9% (w/v) (Frontline Combo®, Merial), and another group treated with a spot-on formulation of dinotefuran 4.95% (w/w), pyriproxyfen 0.44% (w/w) and permethrin 36.08% (w/w) (Vectra 3D®, Ceva) were included in the study 160 days after its commencement. Each of the treated groups and the control group consisted of eight dogs. All dogs were infested on multiple occasions and examined for live parasites at predetermined time intervals after infestation.

The medicated collars were fastened around the dogs’ necks and adjusted until a comfortable fit was achieved. Excess collar was pulled through the collars’ loops and any excess length beyond 2 cm was cut off. The fipronil/(s)-methoprene spot-on formulation was administered at a weight specific dosage of 0.067 ml/kg body weight and applied as a single spot between the shoulder blades. The spot-on formulation of dinotefuran/pyriproxyfen/permethrin was administered at a dosage level of 0.14 ml/kg body weight. The calculated dose was applied at three spots along the dog’s back, namely between the shoulder blades, in the middle of the back and at the base of the tail. The choice of dosage level, dose regimen and of evaluation time-points will be dealt with more extensively in the discussion.

The experimental design of the study is summarized in Table 2.

**Table 2:** **Experimental design of an ectoparasiticidal efficacy study against *Rhipicephalus sanguineus *and *Ctenocephalides felis felis*on dogs, Study 1**

**Study Day**	**Activity**		
**-6**	24 dogs each infested with 100 fleas		
**-5**	Fleas counted and flea numbers used for ranking and allocation to groups		
**-1**	Allocation to control group of 8 dogs (Group 1) and two treated groups (Groups 2, 3)		
**0**	Dogs infested with 100 fleas and 50 ticks immediately prior to collaring		
**2**			48 h flea and tick counts*
**7**		50 ticks	
**8**	100 fleas		
**9**			24 h flea, 48 h tick counts
**14 to 30**	Infestation and counting at weekly intervals the same as for Days 7 to 9 above		
**56**		50 ticks	
**57**	100 fleas		
**58**			24 h flea, 48 h tick counts
**84 to 142**	Infestation and counting at 4-weekly intervals the same as for Days 56 to 58 above		
**160**	Two fresh groups of 8 dogs each (Groups 4 and 5) added to the study		
**161**	Infestation with fleas and ticks (Groups 1, 4 and 5), followed by spot-on treatment of dogs in groups 4 and 5.		
**163**	Flea and tick counts 48 h after treatment on dogs in Groups 1, 4 and 5, thereafter dogs in all five groups subject to the same infestation and counting regimen		
**168**		50 ticks	
**169**	100 fleas		
**170**			24 h flea, 48 h tick counts
**170**	End of label claim for Group 3, no further evaluation		
**175 to 198**	Infestation and counting at weekly intervals the same as for Days 168 to 170 above		
**199**	End of label claim for Groups 4 and 5, no further evaluation		
**224**		50 ticks	
**225**	100 fleas		
**226**			24 h flea, 48 h tick counts

* ticks and fleas counted 48 h after application of the collars.

### Methods: study 2

This study was designed to evaluate the immediate and persistent “repellent” efficacies (measured at 6 h) and “fast killing” efficacies (measured at 18 h) of a collar containing imidacloprid 10.5% (w/w) and flumethrin 4.5% (w/w) (Seresto®, Bayer Animal Health) against *R. sanguineus* and *C. felis felis* on dogs over a period of 71 days. In addition it was so designed that the immediate and persistent efficacy of a spot-on formulation of (s)-methoprene 5.8%/amitraz 7.6%/fipronil 6.4% (w/v) (Certifect®, Merial) administered twice, 35 days apart, could be determined, and also so that the efficacy of a single treatment with the latter remedy on a group of dogs introduced into the study 35 days after its commencement could be determined.

The imidacloprid/flumethrin collars were fastened around the dogs’ necks as previously described. The (s)-methoprene/amitraz/fipronil combination was applied topically on the skin of the neck between the base of the skull and the shoulder blades as two spots of approximately equal volume at a dosage rate of 0.107 ml/kg body weight. Exact weight dependent dosages were chosen for the spot-on product to ensure appropriate comparability with the collar and this will be addressed in greater detail in the discussion.

The study was conducted in two phases. Phase 1 was carried out on 24 dogs allocated to three groups and the dogs in these groups also participated in Phase 2 of the study. In Phase 2 eight fresh dogs were added to the study 35 days after its commencement. The experimental design of the study is summarized in Table 3.

**Table 3:** **Experimental design of an ectoparasiticidal efficacy study against *Rhipicephalus sanguineus *and *Ctenocephalides felis felis *on dogs, Study 2**

**Study day**	**Activity**			
**-5 to -3**	Flea infestation, counting and allocation to 3 groups of 8 dogs (Groups 1, 2, 3)			
**0**	Controls (Group 1) and treated dogs (Groups 2 and 3) infested with fleas and ticks, and treated directly thereafter (Groups 2 and 3)			
**2**			48 h flea + tick counts	
**7**	50 ticks, 100 fleas	6 h tick counts		
**8**			18 h tick counts	24 h flea counts
**14 to 29**	Infestation and counting at weekly intervals the same as for Days 7 and 8 above			
**34**	Fresh group of 8 dogs (Groups 4) added to study			
**35**	Groups 1, 2 and 3 infested with fleas + ticks, Group 3 dogs re-treated and Group 4 dogs treated for the first time directly thereafter			
		6 h tick counts (Groups 1, 2, and 3)		
**36**		18 h tick counts and 24 h flea counts (Groups 1, 2, and 3)		
**42 (all groups)**	50 ticks, 100 fleas	6 h tick counts		
**43**			18 h tick counts	24 h flea counts
**49 to 71**	Infestation and counting at weekly intervals the same as for Days 42 and 43 above			

## Results

### Results: study 1

#### Acaricidal (48 h) efficacy against *R. sanguineus*

The arithmetic mean tick counts of the untreated control group of dogs 48 h after infestation varied between 23.6 and 38.4, thus ensuring a robust challenge with ticks on all assessment days. The tick counts of the dogs in each of the treatment groups are summarized in Table 4.

**Table 4:** **Study 1: Mean numbers of ticks on treated dogs 48 h after treatment and 48 h after each re-infestation with * Rhipicephalus sanguineus***

**Day**	**Mean numbers of ticks (48 h after re-infestation)**				
	**Untreated controls (Group 1)**	**Imidacloprid/** **flumethrin (Group 2)**	**Deltamethrin** **(Group 3)**	**Fipronil/** **(s)-methoprene (Group 4)**	**Dinotefuran/** **pyriproxyfen/** **permethrin (Group 5)**
2*	28.8	6.3	3.9	-	-
**9**	36.0	0.3	4.3	-	-
**16**	33.9	0.8	0.9	-	-
**23**	38.4	0.0	2.8	-	-
**30**	33.4	0.1	3.4	-	-
**58**	26.5	0.1	8.1	-	-
**86**	31.1	0.3	8.3	-	-
**114**	29.1	0.4	2.3	-	-
**142**	33.3	0.0	5.9	-	-
**163**	29.9	-	-	3.3*	6.0*
**170**	31.3	0.0	5.1	0.1	0.6
**177**	33.1	0.1	-	0.5	1.5
**184**	32.8	0.1	-	2.4	2.8
**191**	34.1	0.3	-	2.5	5.5
**198**	23.6	0.0	-	13.4	9.1
**226**	29.4	0.4	-	-	-

* Flea counts 48 h after treatment.

The mean tick counts recorded for the four treated groups of dogs differed significantly (p < 0.05) from those of the untreated control group of dogs (Group 1) on all assessment days. The imidacloprid/flumethrin collared dogs (Group 2) harboured significantly (p < 0.05) fewer ticks 48 h after infestation on Days 58, 86 and 170 than the group of deltamethrin collared dogs (Group 3), significantly (p < 0.05) fewer ticks on Day 198 than the dogs treated with fipronil/(s) methoprene (Group 4), and significantly (p < 0.05) fewer ticks on Days 191 and 198 than the dogs treated with dinotefuran/pyriproxyfen/permethrin (Group 5).

**Figure 1:**
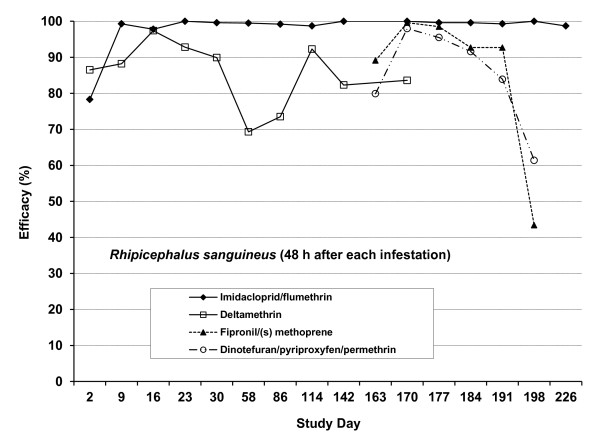
**Study 1.** Efficacy of imidacloprid/flumeththrin and deltamethrin collars, and fipronil/(s) methoprene and dinotefuran/pyriproxyfen/permethrin spot-on formulations against *Rhipicephalus sanguineus* on dogs 48 h after treatment and 48 h after each re-infestation.

The immediate efficacy of the various remedies against ticks 48 h after treatment and persistent efficacy 48 h after each re-infestation are graphically illustrated in Figure 1.

Immediate efficacies were similar on the imidacloprid/flumethrin, deltamethrin, fipronil/(s)-methoprene and dinotefuran/pyriproxyfen/permethrin treated groups of dogs (78.3%, 86.5% 89.1% and 79.9%). Thereafter the persistent efficacies of the imidacloprid/flumethrin collar exceeded 97.8% until the termination of the study on Day 226. Persistent, preventive acaricidal efficacies recorded for this group of dogs were also consistently higher than those recorded for the other treated groups of dogs. With the exception of Days 16, 23 and 114 after infestation, when efficacies above 90% were recorded for the deltamethrin treated group of dogs, persistent efficacies for this group remained below the 90% level. Persistent efficacies exceeding 90% lasted for four weeks on the dogs treated with fipronil/(s)-methoprene, and for three weeks on the dogs treated with dinotefuran/pyriproxyfen/permethrin whereafter efficacy decreased to 43.4% for the fipronil/(s)-methoprene treated group and 61.4% for the dinotefuran/pyriproxyfen/permethrin treated group, five weeks after treatment.

#### Insecticidal (24 h) efficacy against *C. felis felis*

The arithmetic mean flea counts recorded for the untreated control group of dogs varied between 69.6 and 97.1, thus ensuring a robust flea challenge on all assessment days. The mean flea counts of the dogs 48 h after treatment and 24 h after each re-infestation are summarized in Table 5.

**Table 5:** **Study 1: Mean numbers of fleas on treated dogs 48 h after treatment and 24 h after each re-infestation with * Ctenocephalides felis felis***

**Day**	**Mean numbers of fleas (24 h after re-infestation)**				
	**Untreated controls (Group 1)**	**Imidacloprid/** **flumethrin (Group 2)**	**Deltamethrin** **(Group 3)**	**Fipronil/** **(s)-methoprene (Group 4)**	**Dinotefuran/** **pyriproxyfen/** **permethrin (Group 5)**
2*	70.4	0.1	21.4	-	-
**9**	70.1	0.0	15.3	-	-
**16**	78.8	0.0	21.1	-	-
**23**	80.0	0.0	14.3	-	-
**30**	83.8	0.0	17.9	-	-
**58**	69.6	0.0	13.9	-	-
**86**	72.0	0.0	19.5	-	-
**114**	72.5	0.3	24.1	-	-
**142**	72.3	0.4	16.4	-	-
**163**	72.5	-	-	0.0*	0.0*
**170**	78.0	2.8	13.3	0.0	0.0
**177**	88.8	6.0	-	0.0	2.0
**184**	89.1	2.3	-	0.0	1.1
**191**	91.4	5.0	-	1.4	13.3
**198**	97.1	8.9	-	11.1	25.5
**226**	82.6	5.9	-		

* Flea counts 48 h after treatment.

The flea counts recorded for the four treated groups of dogs differed significantly (p < 0.05) from those of the untreated control group (Group 1) on all assessment days. The imidacloprid/flumethrin collared group of dogs (Group 2) had significantly (p < 0.05) fewer fleas 24 h post-infestation than the deltamethrin collared group of dogs (Group 3) on all assessment days except Day 9. No significant (p > 0.05) differences between the arithmetic mean numbers of fleas on the imidacloprid/flumethrin collared, fipronil/(s)-methoprene and dinotefuran/pyriproxyfen/permethrin spot-on treated groups (Groups 4 and 5) were observed.

**Figure 2:**
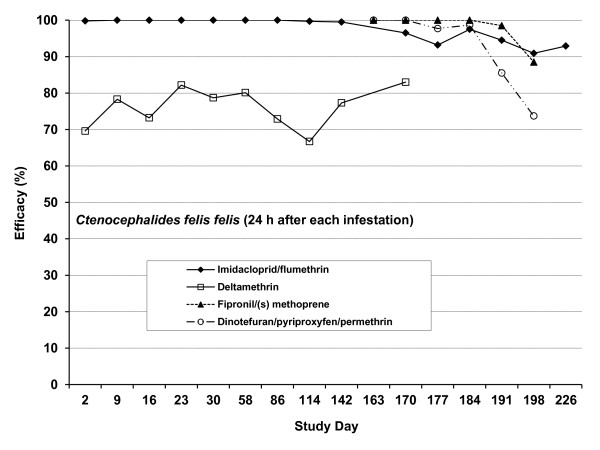
**Study 1.** Efficacy of imidacloprid/flumeththrin and deltamethrin collars, and fipronil/(s) methoprene and dinotefuran/pyriproxyfen/permethrin spot-on formulations against *Ctenocephalides felis felis* on dogs 48 h after treatment and 24 h after each re-infestation.

The immediate efficacy of the various remedies against fleas 48 h after treatment and persistent efficacy 24 h after each re-infestation are graphically illustrated in Figure 2.

The immediate efficacy of the imidacloprid/flumethrin collar against *C. felis felis* 48 h after collaring was 99.8%, and with the exception of Day 177 when an efficacy of 93.2% was recorded, persistent efficacies, assessed 24 h after each re-infestation, varied between 94.5% and 100% until Day 191, decreasing thereafter to 90.9% and 92.9% on Days 198 and 226 respectively. The immediate efficacy of the deltamethrin collars against fleas, assessed 48 h after collaring, was 69.6%, and persistent efficacies, assessed 24 h after each re-infestation, varied between a low of 66.7% on Day 114 and a high of 83.0% on Day 170. The spot-on treatments with fipronil/(s) methoprene and dinotefuran/pyriproxyfen/permethrin resulted in immediate efficacies of 100%. Persistent efficacies greater than 95% were recorded for four weeks on the fipronil/(s) methoprene treated group of dogs and three weeks on the dinotefuran/pyriproxyfen/permethrin group of dogs, thereafter efficacy decreased to 88.5% for the fipronil/(s)-methoprene treated group and 73.7% for the dinotefuran/pyriproxyfen/permethrin treated group, five weeks post treatment.

### Results: study 2

#### “Fast killing” acaricidal (18 h) efficacy against *R. sanguineus*

The arithmetic mean numbers of ticks counted on dogs in the untreated control group 18 h after infestation varied between 20.5 and 36.9, thus ensuring a robust tick challenge on all assessment days. The tick counts of the dogs in each of the treatment groups are summarized in Table 6.

**Table 6:** **Study 2: Mean numbers of ticks on treated dogs 48 h after treatment and 18 h after each re-infestation with * Rhipicephalus sanguineus***

**Day**	**Mean numbers of ticks (18 h after re-infestation)**			
	**Untreated controls (Group 1)**	**Imidacloprid/** **flumethrin** **(Group 2)**	**(S)-methoprene/** **amitraz/fipronil (Group 3)re-treatment: Day 35**	**(S)-methoprene/** **amitraz/fipronil** **(Group 4)**
2*	20.5	4.5	1.6	-
**8**	25.4	0.3	0.0	-
**15**	30.9	0.1	0.6	-
**22**	31.5	0.1	1.9	-
**29**	34.4	0.3	4.6	-
**36**	34.9	0.4	0.5**	-
**43**	32.1	0.4	0.0	0.4
**50**	33.6	0.1	1.0	2.3
**57**	35.8	0.4	2.5	3.9
**64**	36.9	0.8	5.8	12.0
**71**	33.1	0.1	9.0	15.1

* Tick counts 48 h after treatment.

** Tick counts 18 h after re-treatment.

The mean tick counts recorded 18 h after infestation for all treated groups of dogs (Groups 2, 3 and 4) differed significantly (p < 0.05) from those of the untreated control group (Group 1) on all assessment days. The mean tick counts recorded for the imidacloprid/flumethrin treated group were significantly (p < 0.05) lower on Days 29, 64 and 71 than those of the group treated twice with (s)-methoprene/amitraz/fipronil, and also significantly lower on Days 57, 64 and 71 than those of the group of dogs treated for the first time on Day 35.

**Figure 3:**
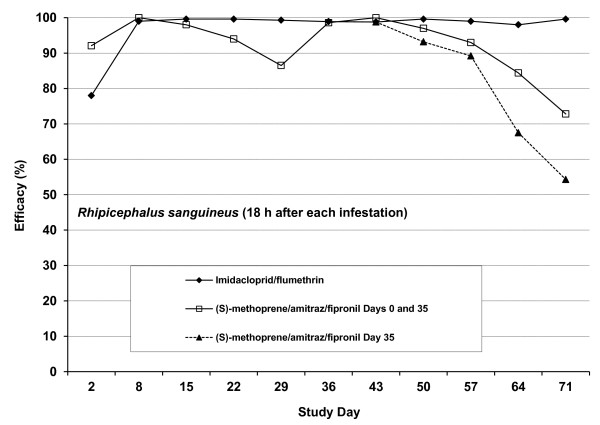
**Study 2.** Efficacy of an imidacloprid/flumeththrin collar and a (s) –methoprene/amitraz/fipronil spot-on formulation against *Rhipicephalus sanguineus* on dogs 48 h after treatment and 18 h after each re-infestation.

The immediate efficacy of the various remedies against ticks 48 h after treatment and persistent efficacy 18 h after each re-infestation are graphically illustrated in Figure 3.

The immediate efficacy of (s)-methoprene/amitraz/fipronil against ticks was markedly superior to that recorded for the imidacloprid/flumethrin collars. However, persistent fast-killing efficacies for the imidacloprid/flumethrin collars assessed 18 h post-infestation was ≥ 98% for the duration of the study, whereas persistent efficacy on the dogs treated twice with (s)-methoprene/amitraz/fipronil exceeded 90% on Days 8, 15 and 22 after the first treatment and on the same days after the second treatment. Persistent tick efficacy on the group of dogs treated with (s)-methoprene/amitraz/fipronil for the first time on Day 35 exceeded 90% in the first two weeks after treatment (Days 43 and 50).

#### “Repellent” or acaricidal (6 h) efficacy against *R. sanguineus*

The arithmetic mean tick counts recorded on the dogs in the untreated control group 6 h after infestation varied between 27.8 and 36.9, thus ensuring a robust tick challenge on all assessment days. The mean tick counts of the dogs in the different treatment groups are summarized in Table 7.

**Table 7:** **Study 2: Mean numbers of ticks on treated dogs 6 h after each infestation with* Rhipicephalus sanguineus***

**Day**	**Mean numbers of ticks (6 h after re-infestation)**			
	**Untreated controls** **(Group 1)**	**Imidacloprid/** **flumethrin** **(Group 2)**	**(S)-methoprene/** **amitraz/fipronil (Group 3)** **re-treatment: Day 35**	**(S)-methoprene/** **amitraz/fipronil** **(Group 4)**
**7**	27.8	4.0	2.1	-
**14**	32.9	2.3	5.8	-
**21**	32.4	1.8	7.4	-
**28**	34.1	1.4	10.0	-
**35**	36.9	1.1	4.0*	-
**42**	30.4	0.9	0.8	1.5
**49**	34.6	1.9	4.4	9.3
**56**	35.3	3.5	8.1	14.8
**63**	36.9	2.5	10.4	12.8
**70**	35.3	1.4	12.3	17.0

* Tick count 6 h after re-treatment.

The tick counts of the dogs in each of the treated groups 6 h after infestation differed significantly (p < 0.05) from those of the untreated control group on all assessment days. The tick counts of the dogs in the imidacloprid/flumethrin treated group were significantly (p < 0.05) lower on Days 28, 56, 67 and 70 than those of the dogs treated twice with (s)-methoprene/amitraz/fipronil, and also significantly (p < 0.05) lower on Days 49, 56, 63 and 70 than those of the dogs in the group treated for the first time on Day 35.

**Figure 4:**
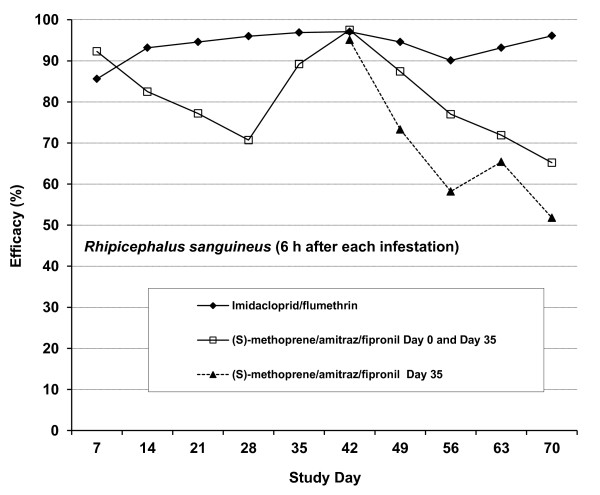
**Study 2.** Efficacy of an imidacloprid/flumeththrin collar and a (s) –methoprene/amitraz/fipronil spot-on formulation against *Rhipicephalus sanguineus* on dogs 6 h after each infestation.

The efficacies of the various treatments against ticks 6 h after each infestation are graphically illustrated in Figure 4.

Except for Days 7 and 42, the persistent efficacies against *R. sanguineus* recorded 6 h after infestation on the group of dogs fitted with imidacloprid/flumethrin collars were consistently higher than those of the groups of dogs treated twice or once with (s)-methoprene/amitraz/fipronil.

#### Insecticidal (24 h) efficacy against *C. felis felis*

The arithmetic mean flea counts of the untreated control group of dogs varied between 69.3 and 85.4, thus ensuring a robust challenge with fleas on all assessment days. The mean flea counts of the dogs in each of the treatment groups are summarized in Table 8.

**Table 8:** **Study 2: Mean numbers of fleas on treated dogs 48 h after treatment and 24 h after each re-infestation with * Ctenocephalides felis felis***

**Day**	**Mean numbers of fleas (24 h after re-infestation)**			
	**Untreated controls (Group 1)**	**Imidacloprid/** **flumethrin** **(Group 2)**	**(S)-methoprene/** **amitraz/fipronil** **(Group 3)** **retreatment: Day 35**	**(S)-methoprene/** **amitraz/fipronil (Group 4)**
2*	75.3	0.3	0.5	-
**8**	69.3	0.0	0.0	-
**15**	83.4	0.0	0.1	-
**22**	80.9	0.0	0.5	-
**29**	85.4	0.0	1.1	-
**36**	73.9	0.4	0.0**	-
**43**	75.0	0.0	0.0	0.0
**50**	74.1	0.0	0.1	0.0
**57**	75.8	0.1	0.8	0.0
**64**	72.5	0.0	1.0	5.9
**71**	85.3	0.4	10.6	17.5

* flea counts 48 h after treatment.

** flea counts 24 h after re-treatment.

The flea counts of the dogs in the treated groups differed significantly (p < 0.05) from those of the untreated control group of dogs (Group 1) on all assessment days. No significant differences (p > 0.05) in the mean flea counts of dogs treated with the imidacloprid/flumethrin collars (Group 2) and treated twice with the methoprene/amitraz/fipronil formulation (Group 3) were observed. The mean flea counts on Day 71 of dogs in the group fitted with imidacloprid/flumethrin collars were significantly (p < 0.05) lower than those of the dogs treated for the first time on Day 35 with (s)-methoprene/amitraz/fipronil (Group 4).

**Figure 5:**
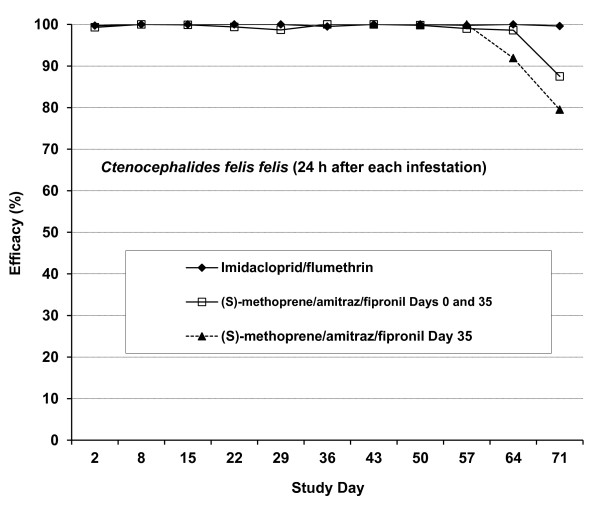
**Study 2.** Efficacy of an imidacloprid/flumeththrin collar and a (s) –methoprene/amitraz/fipronil spot-on formulation against *Ctenocephalides felis felis* on dogs 48 h after treatment and 24 h after each re-infestation.

Efficacy values derived from the 24 h flea counts are graphically illustrated in Figure 5.

Immediate efficacies against fleas recorded for both treatment groups on Day 2 were similar. Persistent efficacies assessed 24 h after re-infestation exceeded 98% in all treated groups up to Day 57. However, efficacy in the group of dogs treated twice with (s)-methoprene/amitraz/fipronil declined from 98.6% on Day 64 (4 weeks after re-treatment) to 87.5% a week later. Efficacy in the group of dogs treated for the first time on Day 35 declined to 91.9% by Day 64 (4 weeks after treatment) and 79.5% a week later. Persistent efficacy in the imidacloprid/flumethrin treated group exceeded 99% for the duration of the study.

## Discussion

### Discussion of study design

When conducting a study comparing the long-term acaricidal efficacy of collars against that of the medium-term efficacy of spot-on products, a number of difficulties arise. These are related to the nature of the remedies, the choice of treatment, the evaluation time-points and the choice of the dose regimen.

#### General arrangement of treatment and comparison time-points

The aim of the study was to give an experimentally-based assessment of the performance over time of a variety of remedies that are available for the treatment and control of ticks and fleas on dogs. Because of the difficulties listed above, a straightforward, direct comparison embracing all treatments and starting on Day 0 would not result in an ideal outcome. However, as both the imidacloprid/flumethrin and the deltamethrin collars claim long-term efficacy of 8 months and 6 months respectively, the decision was taken to start simultaneous treatment with them on Day 0 of Study 1. In addition, because the imidacloprid/flumethrin collar claims efficacy lasting 8 months it would only be fair to compare its efficacy towards the end of this period of time with that of freshly applied spot-on treatments that claim efficacy of four weeks. Consequently treatment with the two spot-on products included in Study 1 was only administered on Day 161 (5 months + 11 days into the study). This allowed for a comparison of efficacy between the imidacloprid/flumethrin collar towards the end of its claimed period of efficacy and those of the two spot-on treatments applied during this time.

The investigative periods for the imidacloprid/flumethrin and the deltamethrin collars comprised the length of time claimed for their respective efficacies. In contrast the investigative periods for the spot-on products was increased by one week beyond their claimed 4-week period of efficacy. This was done to assess the persistent effectiveness of the spot-on treatments should a pet owner decide to extend the period between treatments by a week because of a reliance on the assumed slow decrease in efficacy of these remedies. The latter practice can, however, result in unwanted gaps in efficacy.

The choice of treatment time-points for Study 2 followed a similar rationale. The (s)-methoprene/amitraz/fipronil spot-on and the imidacloprid/flumethrin collar were directly compared from Day 0 and onwards. Then on Day 35, subsequent to the claimed period of efficacy of the spot-on formulation, the same group of dogs was re-treated with the same remedy. This was done to determine whether regular re-treatment would enhance the performance of the spot-on product, and also so that the effectiveness of multiple treatments with the spot-on formulation could be compared with a single application of the collar formulation.

The efficacy claim for the (s)-methoprene/amitraz/fipronil spot-on is 4 weeks, however, its efficacy against ticks and fleas is said to be 5 weeks according to the product’s SPC and package leaflet, namely one treatment prevents further infestation for 5 weeks by ticks and for up to 5 weeks by fleas. On Day 35 a second (s)-methoprene/amitraz/fipronil spot-on treated group of dogs was enrolled. This was done so that the efficacy of this spot-on treatment could be compared with that of the collar that had already been in place for 35 days and also with that of the second treatment with the spot-on administered to the dogs that had been treated 35 days previously. If re-treatment enhanced the efficacy of the spot-on formulation, a series of regular re-treatments would be a more appropriate comparison with the long-term efficacy of the collars.

#### Infestation and evaluation time-points

The infestation and evaluation time-points in Study 1 were chosen in accordance with the products’ label claims and the *Guidelines for the testing and evaluation of the efficacy of antiparasitic substances for the treatment and prevention of tick and flea infestation in dogs and cats;* EMEA/CVMP/005/2000- Rev.2 and current regulatory practice regarding efficacy evaluation, namely flea counts after 24 h and tick counts after 48 h. Because the efficacies against ticks of the remedies under evaluation in Study 2 were expected to be similar at the 48 h assessment time-points, the choice of the tick counting time-point was based on data already published on the efficacy of the (s)-methoprene/amitraz/fipronil spot-on [16], namely “rapid killing” (18 h post-infestation) or “repellence” (6 h post-infestation).

#### Dosage

Exact weight dependent dosages were chosen for the spot-on remedies as this neutralizes the influence of different animal sizes and body weights in a relatively limited sample size, thus making an equitable comparison between spot-on remedies possible. In spot-on formulations the total amount of active ingredient, or ingredients, are immediately released on the treated animal’s skin. This is not so with collar formulations, which rely on the slow-release of active ingredients. Consequently a weight range dosage level was chosen for the collars, which were carefully adjusted to fit each dog’s neck circumference, and any excess length cut off. The release of active ingredients from the collar is, at least in the case of the imidacloprid/flumethrin collar, a “release on demand” and is therefore nearly directly animal-surface and consequently size related. Thus correctly applied imidacloprid/flumethrin collars deliver a daily active ingredient dosage level that is close to being weight dependent [17]. Furthermore, the daily dosage levels calculated from the release-values over 8 months were found to be comparable for dogs and cats of different sizes [17].

### Discussion of results

The efficacy of the imidacloprid/flumethrin collars against *R. sanguineus* and *C. felis felis*, weeks or even months after their application, was as high as that recorded initially for three spot-on formulations administered to separate groups of dogs either at the same time as the collars, or 5 weeks or 5½; months after the collars had been applied. Furthermore, while the efficacies of the spot-on formulations generally decreased during the successive weeks following their application, those of the imidacloprid/flumethrin collars persisted at the same high level. The decrease in efficacy of the spot-on treatments five weeks after their application indicates that re-treatment subsequent to their 4-week claims of effectiveness is advisable. A second treatment with the (s)-methoprene/amitraz/fipronil spot-on formulation did not enhance or prolong its efficacy during the ensuing evaluation period.

Although the immediate acaricidal efficacy of the imidacloprid/flumethrin collars against *R. sanguineus* at the start of the study (Day 2) was below 90% in the two studies reported here, it equalled or exceeded 78%. With the exception of these results, the persistent acaricidal preventative efficacy of these collars either significantly or noticeably exceeded that of the deltamethrin collars at each assessment time-point. On most occasions persistent efficacy also exceeded those of the spot-on formulations of fipronil/(s)-methoprene and dinotefuran/pyriproxyfen/permethrin, as well as those measured 18 h after infestation, for the (s)-methoprene/amitraz/fipronil spot-on formulation.

Unexpectedly, considerable variation in the acaricidal effectiveness of the deltamethrin collar between individual dogs was observed. This variability contributed to the overall lower than anticipated efficacy of this collar compared to published results on its effectiveness 48 h after re-infestation [18]. In contrast there appeared to be no marked variability in the efficacy of the imidacloprid/flumethrin collars in the present and other studies [19,20].

Repellent effects are a general property of pyrethroids. Their in-contact efficacy comprises a mixture of a very rapid lethal effect, a knock down effect and the so-called “hot-foot effect”, instead of a vapour-based classical repellent effect. Besides the well-described difference of specific ectoparasiticidal potency amongst different pyrethroids of even the same chemical subclass [e.g. Mendes [21] described a 125-fold and 400-fold higher efficacy (EC_50_ after 5 min contact) of flumethrin on *Boophilus microplus* than deltamethrin and cyfluthrin, respectively], the expression of efficacy can also vary within the same molecule. There is a dose-time-dependency for lethal effects in pyrethroids [22] so that the presence of a very fast acaricidal “repellent” effect is perhaps an indication for higher doses on the hair coat when compared to the dose of the same pyrethroid that only results in an acaricidal effect 48 h after its application. In the case of the deltamethrin collar, for which 24 h efficacy against ticks does not exceed the 90% threshold [18], the hair coat concentration of deltamethrin released by the collar is apparently not sufficient to cause a rapid “repellent”-type efficacy. In contrast the hair coat concentrations after application of the imidacloprid/flumethrin collar obviously exceed the critical dose required to achieve 18 h and even 6 h efficacy. This suggests that the 48 h acaricidal efficacy of the latter collar is backed by a resilient safety margin of active ingredient on the animal’s hair coat.

The long-term repellent efficacy of the imidacloprid/flumethrin collars in Study 2, measured 6 h after infestation with *R. sanguineus*, was, with the exception of Day 7 after treatment, above 90%, followed by “rapid killing” acaricidal efficacies ≥98% against the same population of ticks 12 h later (Tables 6 and 7). With the exception of Days 7 and 42, when the 6 h repellent efficacy of the (s)-methoprene/amitraz/fipronil spot-on treatment exceeded that of the collars, and Days 8 and 43 when the 18 h “rapid killing” efficacy of the spot-on treatment exceeded that of the collars, the effectiveness of the collars exceeded that of the spot-on formulation. The rapidity with which ticks are killed by the active ingredients of the collars and the spot-on treatment, implies that there might be significant interference with the transmission of tick-borne organisms.

The excellent immediate and medium-term efficacies of the spot-on formulations of (s)-methoprene/fipronil, dinotefuran/pyriproxyfen/permethrin and (s)-methoprene/amitraz/fipronil against *C. felis felis* makes them suitable candidates for the immediate and medium-term control of fleas and hence also flea allergic dermatitis on dogs [6]. In addition to its immediate high efficacy against fleas, the approximately 8-month long persistent efficacy of the imidacloprid/flumethrin collar should control fleas for the whole flea season from late winter to autumn. The collars could thus also constitute an important component in the multi-remedy regimen required for the treatment and prevention of flea allergy dermatitis during the entire season of flea activity [6].

Although the effectiveness of the various remedies tested in these studies may at various stages after their application be excellent, it is the number of living ticks or fleas remaining on treated dogs, when efficacy decreases or is poor, that are important. Tick burdens exceeding 10 individuals after treatment are quite adequate for the transmission or acquisition of organisms responsible for tick-borne diseases in the field, while a few fleas remaining after ineffective treatment are capable of inducing severe signs of flea allergy dermatitis. This implies that if pet owners do not comply with the recommended time-periods between the administration of spot-on remedies serious gaps in efficacy may occur. The long-term, persistently high efficacy of the imidacloprid/flumetrin collars would appear to be an excellent counter to these eventualities.

The studies above were all laboratory based, while in the field various challenges to the efficacy of the medicated collars may occur. The most obvious of these is that dogs are inevitably going to be washed or shampooed, or swim or go out into the rain while being walked by their owners or working in the field. Importantly collars do not have to be removed during any of these events [20], and that their efficacy against re-infestation with *R. sanguineus* remained above 97% over a period of 8 months on regularly shampooed dogs, and above 94% on dogs regularly immersed in water. Efficacy against *C. felis felis* remained above 90% on shampooed dogs for the 8-month duration of the study, but declined to below 90% at 6 months in the group of dogs that were regularly immersed in water [20].

## Conclusions

The 8-month long period of efficacy of medicated collars incorporating a combination of 10% imidacloprid and 4.5% flumethrin against repeated infestations of *Rhipicephalus sanguineus* and *Ctenocephalides felis felis* on dogs, provides the wherewithal to overcome the fluctuating medium-term efficacy of spot-on treatments resulting from a lack of pet owner re-treatment compliance. The sustained high level of efficacy of the collars against ticks 6 hours and fleas 24 hours after infestation, may well interfere with the transmission of vector-borne diseases.

## Competing interests

These clinical studies were completely funded by Bayer Animal Health GmbH, Monheim, Germany, of which D. Stanneck (Germany) is an employee. ClinVet is an independent Contract Development Organisation, which was contracted to manage the conduct of both studies. I.G. Horak is a long-term, contract employee of Clinvet and an Extraordinary Professor at the Universities of the Free State and Pretoria. All authors voluntarily publish this article and have no personal interest in these studies other than publishing the scientific findings that they have been involved in via planning, setting-up, monitoring and conducting the investigations and analysing the results.

## Author’s contributions

DS and JJF designed the study and drafted the protocol and JJF conducted the study. IGH compiled and analysed the data and wrote the initial drafts of the manuscript. All authors read and approved the final manuscript.
